# A Comparative Study of the First and Second Waves of COVID-19 in Hemodialysis Patients From Pakistan

**DOI:** 10.7759/cureus.21512

**Published:** 2022-01-23

**Authors:** Shabana Rahim, Murtaza Dhrolia, Ruqaya Qureshi, Kiran Nasir, Aasim Ahmad

**Affiliations:** 1 Nephrology, The Kidney Centre Post Graduate Training Institute, Karachi, PAK

**Keywords:** hemodialysis, covid-19, outcome, characteristics, first and second waves

## Abstract

Introduction: This study aims to compare the characteristics and outcomes of the first and second waves of coronavirus disease 2019 (COVID-19) in hemodialysis (HD) patients.

Method: We compared the epidemiological, clinical, laboratory, and radiological characteristics and outcomes of a cohort of HD patients who contracted COVID-19 in our HD center during the first wave from May 2020 to September 2020 and the second wave from November 2020 to February 2021.

Results: A total of 50 (11.8%) of 423 patients during the first wave and 46 (10.5%) of 437 patients during the second wave contracted COVID-19. The median age was 59.5 ± 9.99 years (first wave) and 60.3 ± 13.02 years (second wave). Most patients developed the mild disease. Patients requiring hospitalization (22% vs. 32.6%) and mechanical ventilation (10% vs. 17.4%) were more in the second wave. The most common symptom was fever (82% and 63%) in both waves. Patchy bilateral opacity was the most common radiological finding. Major complications including lymphocytopenia (36% and 63%), pneumonia (28% and 32.6%), thrombocytopenia (30% and 17.4%), and septic shock (6% and 10.9%) were shared. Ten (20%) patients died in the first wave and 13 (28.3%) in the second wave. Patients aged > 60 years had more severe disease and died more than patients aged < 60 years in both waves.

Conclusion: There is a high susceptibility and mortality of HD patients in both the first and second waves of COVID-19 as compared to the general population. Disease symptoms, radiological findings, and laboratory tests were similar in both waves. Patients developing critical disease and requiring hospitalization and mechanical ventilation were more in the second wave.

## Introduction

The sudden emergence of the coronavirus disease 2019 (COVID-19) pandemic raised serious health threats globally with devastating outcomes. The COVID-19 struck in waves, and many countries saw a second wave of the COVID-19 during 2020 and 2021. Unavailability of specific antivirals or vaccines even till the emergence of the second wave necessitated non-pharmaceutical interventions as the benchmark in restricting the spread of severe acute respiratory syndrome coronavirus 2 (SARS-CoV-2). Non-compliance to these precautions, such as physical distancing, hand-washing, and mask-wearing was the apparent cause of the second wave of COVID-19 [1,2].

In Pakistan, the first case of COVID-19 was reported on 26th February 2020. The first wave infected more than 300,000, claimed many lives, and affected millions of people socio-economically [3]. The first wave peaked in June 2020, and then the number of new cases/day drastically come down [3,4]. However, with the gradual easing of the lockdown and opening of many social, political, religious, and regular business activities, the number of COVID-19 cases started climbing again, and in Pakistan, the government announced a second wave of COVID-19 on October 28, 2020 [5]. The data released by the National Command and Operation Centre (NCOC) indicated that the percent positivity rate and death rate of the second wave were higher than that of the first wave [1,6].

Considering the large population size of hemodialysis (HD) patients [7], the compromised immune function of uremic patients [8] along with the increased frequency of comorbidities such as diabetes, hypertension, and cardiovascular disease among maintenance hemodialysis (MHD) patients, it was anticipated even at the arrival of the first COVID-19 wave that MHD patients were likely to be more susceptible to COVID-19 infection but also to have severe illness and higher mortality risk than that for the general population [9-11]. The clinical characteristics and outcomes of COVID-19 were diverse in this group, ranging from asymptomatic to deadly [9-12], making the trend of the illness in individual cases unpredictable. The second wave was not only linked to new variants of the SARS-CoV-2, but it also differed in factors such as age range and severity of the disease [13,14].

## Materials and methods

This is a retrospective, comparative, cohort study. We reviewed the epidemiological, clinical, laboratory, and radiological characteristics and outcomes of a cohort of HD patients who contracted COVID-19 in our HD center during the first and second waves of COVID-19, after obtaining approval from the institutional ethical review committee and informed consent from participants. Information collected included demographics, exposure history, dialysis vintage, comorbidities, symptoms, signs, radiological and laboratory tests, complications, and treatment received during COVID-19 infection and outcome.

A confirmed case of COVID-19 was defined as a positive result on at least one of two tests done 24 hours apart on real-time reverse transcription-polymerase chain reaction (RT-PCR) assay of nasal swab specimens [15]. The incubation period was defined as the interval between the earliest date of likely contact of the transmission source (person with suspected or confirmed case) and the earliest date of symptom onset. Lymphocytopenia was defined as a lymphocyte count of less than 1,500 cells per cubic millimeter. Thrombocytopenia was defined as a platelet count of less than 150,000 per cubic millimeter. Pneumonia was diagnosed using the American Thoracic Society guidelines for community-acquired pneumonia [16], septic shock was diagnosed using the Third International Consensus Definitions for Sepsis and Septic Shock [17], disseminated intravascular coagulation (DIC) was diagnosed according to the International Society on Thrombosis and Haemostasis (ISTH) criteria [18], and acute respiratory distress syndrome (ARDS) was defined according to the Berlin definition [19]. Acute hepatic injury was defined as an elevation in alanine aminotransferase of more than 10 times the upper limit of normal [20].

We defined the degree of severity of COVID-19 as mild, moderate-severe, and critical. Mild refers to patients who had mild symptoms without manifestation of viral pneumonia on the chest X-ray. Moderate-severe disease refers to patients who had symptoms such as fever and respiratory tract symptoms, with features of viral pneumonia on the chest X-ray with or without respiratory rate >30 breaths/min, oxygen saturation <93% at rest state, and pulmonary lesion progression of >50% within 24-48 hours on radiologic imaging. Critical refers to patients with respiratory failure requiring mechanical ventilation, and/or with the presence of shock, and/or another organ failure that requires monitoring and treatment in the high dependency unit (HDU) or intensive care unit (ICU).

Statistical analysis

Data were entered and analyzed by SPSS version 21 (IBM Corp., Armonk, NY). Mean with standard deviation and median with interquartile ratio (IQR) were calculated for continuous variables, while for categorical variables, frequencies with percentage were obtained. Logistic regression was run to observe the effect of different variables on the severity of disease and the outcome of COVID-19, and the odds ratio with 95% CI was obtained. For binary logistic regression, we merged the three categories of disease severity into two categories, i.e., severe and non-severe. The death rate associated with COVID-19 in HD patients was compared with deaths in HD patients during the same period in the previous year. A P-value of less than or equal to 0.05 was considered.

## Results

A total of 423 MHD patients were registered at our center during the first wave and 437 during the second wave. Fifty patients (11.8%) during the first wave and 46 patients (10.5%) during the second were diagnosed as confirmed cases of COVID-19.

In the first wave, affected males were 26 (52%) and females were 24 (48%), whereas, in the second wave, there were 29 (63%) males and 17 (37%) females. The median age was similar in both waves: 59.5 ± 9.99 years (first wave) and 60.3 ± 13.02 (second wave). The patients who were above 60 years of age were 30 (60%) in the first wave and 28 (60.9%) in the second wave. The major cause of chronic kidney disease was diabetes mellitus (DM) in 22 (44%) patients followed by hypertension (HTN) in 11 (22%) patients in the first wave and unknown cause in 18 (39.1%) patients followed by DM in eight (17.4%) patients in the second wave. The mean hemodialysis vintage was 4.59 ± 4.7 years in the first wave and 4.36 ± 3.47 years in the second wave. All patients had one or more coexisting morbidities. The most common comorbidities were similar in both waves, HTN being 94% vs. 91.3% followed by diabetes (44% vs. 52.2%) and coronary artery disease (CAD) (36% vs. 23.9%). Exposure history was present in eight (16%) patients (all with the confirmed cases) in the first wave and eight (17.4%) patients in the second wave. The travel history to a high prevalence of COVID-19-infected areas within the country was present in two (4%) patients in the first wave and one (2.2%) patient in the second wave.

Out of 50 patients in the first wave, three patients remained asymptomatic throughout the course of their illness, while in the second wave, four patients out of 46 were asymptomatic. The median incubation period was five days and 4.5 days in the first and the second wave, respectively. Patients who developed the mild disease were 34 (68%) in the first and 27 (58.7%) in the second wave. Patients with moderate-severe disease in the first and the second waves were six (12%) and four (8.7%), respectively, while 10 (20%) in the first wave and 15 (32.6%) in the second wave were critical (p = 0.16). The most common symptoms in the first wave were fever (41, 82%), followed by fatigue (32, 64%), cough (19, 38%), and myalgia/arthralgia (15, 30%), while in the second wave, the most common symptoms were fever (29, 63%), followed by cough (27, 58.7%), shortness of breath (18, 39.1%), and diarrhea (15, 32.6%).

Of patients, 24% in the first wave and 13.1% patients in the second wave had abnormal findings on chest X-rays. The most common pattern was patchy bilateral opacities (ground-glass opacities) in both waves. Detailed demographic, clinical, laboratory, and radiological findings of study participants are given in Table 1.

**Table 1:** Comparison of demographic, clinical, laboratory, and radiological findings of hemodialysis patients with COVID-19 between the first and second waves. ESKD, end-stage kidney disease; ADPKD, adult polycystic kidney disease; BMI, body mass index; AVF, arteriovenous fistula; AV, arteriovenous; BCG, bacillus Calmette-Guérin; LN, lymph node, BP, blood pressure; HB, hemoglobin; WBC, white blood cells; PTH, parathyroid hormone.

Variables	First wave (n = 50)	Second wave (n = 46)
	Mean ± SD, Median, IQR - n (%)	
Gender		
Male	26 (52%)	29 (63%)
Female	24 (48%)	17 (37%)
Age	59.5 ± 10, 60.5, 11	60.3 ± 13.02, 61.5, 15.25
<60 years (n, %)	20 (36%)	18 (39.1%)
>= 60 years (n, %)	30 (60%)	28 (60.9%)
Smoking history (%)		
Never smoked	35 (70%)	36 (78.3%)
Former smoker	12 (24%)	8 (17.4%)
Current smoker	3 (6%)	2 (4.3%)
Exposure to a source of transmission within past 14days	8 (16%)	8 (17.4%)
Living in the same house of COVID-19 patient	4 (8%)	7 (15.2)
Having face-to-face contact	4 (8%)	1 (2.2%)
Contact with a healthcare worker	0 (0%)	0(0%)
History of past 14 days travel in COVID-19-infected area	2 (4%)	1 (2.2%)
Median incubation period	5	4.5
Cause of ESRD		
Unknown	5 (10%)	18 (39.1%)
Diabetes	22 (44%)	8 (17.4%)
Hypertension	11 (22%)	7 (15.2%)
Glomerulonephritis	4 (8%)	4 (8.7%)
ADPKD	4 (8%)	1 (2.2%)
Other	4 (8%)	8 (17.4%)
Comorbidities		
Diabetes	24 (48%)	24 (52.2%)
Hypertension	47 (94%)	42 (91.3%)
Coronary artery disease	18 (36%)	11 (23.9%)
Congestive heart failure	3 (6%)	5 (10.9%)
Pulmonary disease	4 (8%)	4 (8.7%)
Hepatitis B or C	5 (10%)	3 (%)
Obesity (BMI > 30)	10 (20%)	10 (21.7%)
History of renal transplantation in past	3 (6%)	0 (0%)
Hemodialysis vintage	4.59 ± 4.7, 3, 4.25	4.36 ± 3.47, 4.0, 4.0
<5 years (n + %)	32 (64%)	28 (60.9%)
>= 5 years (n + %)	18 (36%)	18 (39.1%)
Access for hemodialysis		
AVF	42 (84%)	44 (95.7%)
AV graft	4 (8%)	0 (0%)
Permacath	4( 8%)	2 (4.3%)
Dialyzer type		
Single-use	19 (38%)	22 (47.8%)
Re-use	31 (62%)	24 (52.2%)
Vaccination		
Flu vaccine	24 (48%)	31 (67.4%)
BCG vaccine	43 (86%)	39 (84.8%)
Symptoms (%)		
Fever	41 (82%)	29 (63%)
Chills	13 (26%)	6 (13%)
Fatigue	32 (64%)	13 (28.3%)
Myalgia/arthralgia	15 (30%)	7 (15.2%)
Cough	19 (38%)	27 (58.7%)
Sore throat	10 (20%)	1 (2.2%)
Shortness of breath	14 (28%)	18 (39.1%)
Sputum production	10 (20%)	0 (0%)
Hemoptysis	0 (0%)	1 (2.2%)
Conjunctival congestion	1 (2%)	0 (0%)
Nasal congestion	5 (10%)	1 (2.2%)
Headache	9 (18%)	4 (8.7%)
Nausea/vomiting	10 (20%)	6 (13%)
Diarrhea	14 (28%)	15 (32.6%)
Signs		
Throat congestion	3 (6%)	1 (2.2%)
Tonsil swelling	0 (0%)	1 (2.2%)
Rash	0 (0%)	0 (0%)
Enlargement of LN	0 (0%)	0 (0%)
Respiratory rate/min	20.9 ± 3, 20, 6	21.1 ± 3.77, 20, 6.0
Heart rate/min	80.6 ± 8, 81, 13.5	84.3 ± 15.6, 85, 19
BP systolic (mmHg)	135.7 ± -22, 140, 37.75	142.5 ± 24.7, 141, 37
BP diastolic (mmHg)	72.8 ± 12, 77.5,12.5	74.0 ± 14.5, 70.0, 20.75
Laboratory tests (mean ± SD, median, IQR)		
HB (g/dl)	10.2 ± 1, 10.1, 1.90	10.1 ± 1.66, 10.1, 2.32
WBC per mm^3^	7.8 ± 4, 7.0, 4.27	8.0 ± 3.08, 7.85, 2.8
Lymphocyte count per mm^3^	2259 ± 2593, 1780.6, 1238.4	1316 ± 563.1, 1367, 718.5
Neutrophil count per mm^3^	5331 ± 3711, 4118, 3872	5895.7 ± 3085.3, 5197, 2639.8
Platelet count per mm^3^	217 ± 106, 186, 131.5	248.1 ± 100.7, 228.5, 103.7
C-reactive protein (mg/l)	13.7 ± 21.9, 6, 0	74 ± 179.1, 24, 85.5
Transferrin saturation (%)	36.3 ± 16, 33.3, 19.7	34.5 ± 13.97, 32.24, 17.61
Ferritin	1468 ± 32755, 790, 903	1472.9 ± 1618, 1315.8, 1426.7
Serum albumin (mg/dl)	3.42 ± 0.5, 3.4, 0.69	3.54 ± 0.52, 3.64, 0.79
Alanine aminotransferase	127 ± 687, 12.5, 12.5	28.3 ± 67, 12, 11
Intact PTH (pg/ml)	381 ± 405, 266.4, 357.48	469.7 ± 575.1, 286.1, 487.3
Radiological findings (chest X-ray)		
Normal	38 (76%)	31 (67.4%)
Local patchy opacity	1 (2%)	0 (0%)
Bilateral patchy opacity	10 (20%)	6 (13.1%)
Interstitial opacity	1 (2%)	0 (0%)

Pneumonia in 14 (28%) vs. 15 (32.6%), septic shock in three (6%) vs. five (10.9%), lymphocytopenia in 18 (36%) vs. 29 (63%), and thrombocytopenia in 15 (30%) vs. eight (17.4%) patients were present, respectively. Oxygen and IV antibiotics were given to 10 (20%) patients in the first wave and 14 (30.4%) patients in the second wave. Four (8%) vs. 14 (30.4%) patients received systemic steroids in the first vs. the second wave (p = 0.005). The number of patients hospitalized was 11 (22%) vs. 15 (32.6%) (p = 0.24), and patients who required mechanical ventilation were five (10%) vs. eight (17.4%) (p = 0.29) in the first and the second wave, respectively. The total number of patients who died was 10 (20%) in the first wave and 13 (28.3%) in the second wave (p = 0.34; Table 2). In the first and the second waves, survival at week one from the date of a positive SARS-CoV-2 test was 98% vs. 93.5%, and survival at week two was 86% vs. 80.4%, respectively. The mean time of death from the date of positive SARS-CoV-2 polymerase chain reaction (PCR) test was 11.9 + 5.8 days in the first wave and 9.76 ± 7.4 in the second wave while from the date of onset of symptoms was 15.2 + 4.96 days in the first wave and 12.3 ± 7.31 in the second wave.

**Table 2:** Comparison of Disease severity, Complications, Treatment used & Outcome of dialysis patients with COVID-19 between first and second wave DIC, disseminated intravascular coagulation; IV, intravenous; HCQ, hydroxychloroquine, ICU, intensive care unit; HDU, high dependency unit.

Variables	First wave (n = 50)	Second wave (n = 46)
	Mean ± SD, median, IQR - n (%)	
Disease severity		
Mild	34 (68%)	27 (58.7%)
Moderate-severe	06 (12%)	4 (8.7%)
Critical	10 (20%)	15 (32.6%)
Complications		
Lymphocytopenia	18 (36%)	29 (63%)
Thrombocytopenia	15( 30%)	8 (17.4%)
Pneumonia	14 (28%)	15 (32.6%)
Acute hepatic injury	3( 6%)	1 (2.2%)
Septic shock	3 (6%)	5 (10.9%)
DIC	0 (0%)	0 (0%)
Acute respiratory distress syndrome	0 (0%)	2 (4.3%)
Treatment used		
Oxygen therapy	10 (20%)	14 (30.4%)
IV antibiotics	10 (20%	14 (30.4%)
HCQ	1 (2%)	1 (2.2%)
Remdesivir	1 (2%)	6 (13%)
Tocilizumab	0 (0%)	1 (%)
Systemic glucocorticoids	4 (8%)	14 (30.4%)
IV immunoglobulin	0 (0%)	0 (0%)
Convalescent plasma	0 (0%)	0 (0%)
Outcome		
Hospitalization	11 (22%)	15 (32.6%)
Isolation ward	2 (4%)	6 (13%)
ICU	6 12%)	6 (13%)
HDU	3 (6%)	3 (6.5%)
Mechanical ventilation	5 (10%)	8 (17.4%)
Invasive	4 (8%)	3 (6.5%)
Non-invasive	1 (2%)	5 (10.9%)
Recovery	40 (80%)	33 (71.7%)
Time from the date of positive test (days)	30.6 ± 14, 29, 20	11.39 ± 4.85,13,7
Time from the date of onset of symptoms (days)	34.4 ± 15, 30.5, 18.7,	14.03 ± -5.2,14,6
Death	10(20%)	13 (28.3%)
Time from the date of positive test (days)	11.9 ± 6, 11, 10	9.76 ± 7.4,6,10.5
Time from the date of onset of symptoms (days)	15.2 ± 5, 13.5, 9.5	12.3 ± 7.31,10,10.5
Death rate with COVID-19 than general	1.46	1.54

The patients aged 60 years and above had 4.3 times more severe disease than patients with age less than 60 years in the first wave (P = 0.044) and in the second wave, it was 6.8 times more severe (P = 0.023). Gender and comorbidities such as DM, HTN, CAD, and obesity did not show significant relation with the severity of disease in both waves. The patients vaccinated with flu vaccine suffered 3.6 times more with severe disease than patients without vaccination (P = 0.049) in the first wave, while in the second wave, flu vaccination had no effect on disease severity (Table 3).

**Table 3:** Comparison of association of study variables with disease severity between the first and second waves. CAD, coronary artery disease; BMI, body mass index; HD, hemodialysis; BCG, bacillus Calmette-Guérin.

Variables	First wave			Second wave		
	Univariate regression analysis			Univariate regression analysis		
	Odds ratio	CI (lower-upper)	P-value	Odds ratio	CI (lower-upper)	P-value
Age						
<60 years	1			1		
>= 60 years	4.3	1.04-1.8	0.044	6.8	1.3-35.4	0.023
Gender						
Female	1			1		
Male	2.25	0.71-7.14	0.169	1.1	0.8-4	0.908
Diabetes	1.63	0.49-5.4	0.425	3.2	0.8-12.4	0.091
CAD	0.73	0.21-2.6	0.63	4.5	0.97-16.8	0.054
Obesity (BMI > 30)	1.6	0.37-6.53	0.55	0.97	0.21-4.5	0.973
HD vintage						
<5 years	1			1		
>= 5 years	1.1	0.2-4.3	0.91	1.9	0.53-6.8	0.321
Dialyzer use						
Single-use	1			1		
Re-use	0.48	0.14-1.6	0.234	0.58	0.16-2.1	0.405
Vaccination						
Flu vaccine	3.6	1.01-12.57	0.049	2.2	0.51-9.5	0.291
BCG vaccine	1.2	0.21-7.0	0.83	0.52	0.1-2.7	0.443

The patients aged 60 years and above died 3.3 times more than patients less than 60 years of age in the first wave, although it was not statistically significant (P = 0.164), as compared to the second wave, 5.8 times more patients died who were 60 years or above. Overall deaths during the first and second waves in HD patients were 1.46 and 1.54 times higher, respectively, than in the same period before the COVID-19 pandemic.

Among all comorbidities, patients with DM in both waves were associated with higher death rates but this was not statistically significant (Table 4).

**Table 4:** Comparison of association of study variables with outcome between the first and second waves. CAD, coronary artery disease; HD hemodialysis; BCG, bacillus Calmette-Guérin.

Variables	First wave			Second wave		
	Univariate regression analysis			Univariate regression analysis		
	Odds ratio	CI (lower-upper)	P-value	Odds ratio	CI (lower-upper)	P-value
Age						
<60 years	1			1		
>= 60 years	3.3	0.62-17.4	0.164	6.8	1.1-30.6	0.036
Gender						
Female	1			1		
Male	0.32	0.07-1.4	0.131	1.1	0.29-4.1	0.894
Diabetes	3.16	0.71-14.0	0.13	4.5	1.1-19.5	0.043
CAD	0.71	0.16-3.19	0.659	4.8	1.1-20.4	0.034
Obesity (BMI > 30)	1	0.17-5.7	0.99	1.1	0.24-5.2	0.89
HD vintage						
<5 years	1			1		
>= 5 years	0.75	0.14-4.1	0.74	1.5	0.41-5.5	0.541
Dialyzer use						
Single-use	1			1		
Re-use	0.32	0.77-1.34	0.119	0.71	0.2-2.6	0.609
Vaccination						
Flu vaccine	3.2	0.71-14.1	0.131	1.9	0.44-8.3	0.391
BCG vaccine	1.6	0.17-14.9	0.686	0.46	0.09-2.4	0.359

## Discussion

The first wave of COVID-19 was particularly devastating for HD patients worldwide, with mortality ranging from 21% to 32.8% in different studies [10,11,21,22], much higher than that of the general population [11,21]. Many countries have gone through the second wave possibly linked to new variants of the SARS-CoV-2. Empirical data suggest that it also differs in factors such as age range and severity of the disease [13,14]. Nephrologists are responsible for counteracting SARS-CoV-2 outbreaks in HD facilities. The lack of similarities between the characteristics of the two waves in HD patients made it imperative to analyze our experience of COVID-19 infection during the first and second waves to improve procedures for screening, managing, and treating dialysis patients affected by COVID-19.

Our study found high susceptibility of HD patients to COVID-19 during the first (11.8%) and the second wave (10.5%) compared to the general population of Pakistan where the positivity rate varied from 3.5% to 8% during our study period [3]. High susceptibility of HD patients was also found in reports from China [9,10] and Italy [11], mainly attributed to the compromised immune status of uremic patients [8], along with the increased frequency of comorbidities in HD patients.

In our study, exposure history was positive in only 16% (all with the confirmed cases) of patients in the first wave and 17.4% in the second wave, possibly suggesting that the primary source of COVID-19 spread may be asymptomatic patients or patients in the incubation period [23] and close contact with other individuals in the HD center. In our dialysis center, all patients are checked for temperature twice, and before starting their session, patients were asked about symptoms related to COVID-19. Those suspected of COVID-19 were then dialyzed in a separate area at different times from the usual shift to minimize interaction with other patients. These suspected and confirmed cases had to have two negative PCR tests before returning to dialysis at their original days and times. All COVID-19 confirmed patients were also dialyzed in a separate area and on different days. Even after all these measures, preventing cross-contamination remained highly challenging during the second wave indicating that we still lack optimal screening and managing approach for HD patients and there is a need for more restrictive screening criteria to combat this problem.

Our data showed that most (60%) HD patients who contracted COVID-19 were older than 60 years of age with a mean age of 59.5 ± 9.99 years (first wave) and 60.3 ± 13.02 years (second wave). This correlates with the findings of studies from Italy [11] and China [9] in HD patients but is significantly higher than the general population (43.2 ± 5.7 years) both during the first [24] and the second wave [25]. Our study also showed that the patients aged 60 years or more had 4.3 times (first wave) and 6.8 times (second wave) more severe disease and 3.3 times (first wave) and 5.8 times (second wave) more death than patients with age less than 60 years. This is similar to findings in the general population during the first [26] and the second wave [27] that adults over 65 years of age represent the majority of hospitalizations and have a higher risk of death than those under 65 years of age.

According to the published literature, COVID-19 patients with underlying conditions such as DM, HTN, cardiovascular disease, or obesity are highly susceptible and often have the more serious disease [28]. However, our study found no significant relationship between comorbidities such as DM, HTN, CAD, and obesity with the severity of the disease in both waves. Comorbidity such as DM and CAD were found to be associated with the higher death rates in univariate analysis in the second wave; however, their association was not found statistically significant in multivariate analysis, possibly because of a small sample size.

The patients vaccinated with the flu vaccine suffered 3.6 times more with severe disease than patients without vaccination (P = 0.049) in the first wave, while in the second wave, flu vaccination had no effect on disease severity in our study patients. Result of studies from Italy [29] and according to early scientific research belief [30], higher influenza vaccination rates were associated with fewer deaths from COVID‐19 in elderly or hospital workers who got vaccinated as they were significantly less likely to develop COVID than those who did not. The negative relation between the flu vaccine and frequency of disease severity and death in our study participants during the first wave is still unexplainable. Gender, HD vintage, type of dialyzer used, vaccination with bacillus Calmette-Guérin (BCG) vaccine, and other laboratory parameters such as lymphocytopenia, C-reactive protein, and albumin showed no significant relation with disease severity or outcome.

Our patients' symptoms and complications were more or less similar in both waves and also correlate with the most frequent signs and symptoms in other studies [9,11,27]. The radiological finding on chest X-ray was bilateral patchy bilateral opacity (ground-glass opacity), which is similar to findings in other studies during the first wave [9,11].

Our study found that the number of patients who developed critical disease was more in the second wave (32.6%) than in the first wave (20%), although not statistically significant. Contrary to the result of our study, a study from Spain [25] found that in the general population, the proportion of patients with mild or severe symptoms compared with those without symptoms or with minor symptoms was significantly higher during the first wave compared with the second wave.

Similarly, in our study, the number of patients requiring hospitalization (22% vs. 32.6%) and mechanical ventilation (10% vs. 17.4%) were more in the second wave (although statistically not significant), while a study from Spain [27] in the general population observed that the second wave caused a significantly fewer number of admissions to internal medicine and ICU, with a shorter duration of hospitalization. Iftimie et al. [27] further noticed that the patients in the second wave were treated more often with non-invasive mechanical ventilation and steroids, and less often with invasive mechanical ventilation, conventional oxygen therapy, and anticoagulants. This is also found in our study as only 8% of patients received systemic steroids during the first wave as compared to 30.4% of patients during the second wave (P = 0.005). Also, non-invasive mechanical ventilation remained the preferred method during the second wave as compared to the first wave.

Our study has certain limitations, some of which have already been discussed above at relevant places including small sample size and non-availability of chest CT. Despite these limitations, this is the first study from Pakistan or any developing country comparing the epidemiologic and clinical characteristics and outcomes of patients undergoing HD between the first and second wave of COVID-19. Some of the important findings reported in our study may help understand the similarities and differences of the two waves of COVID-19 and to make policies for controlling cross-infection and overall management of HD patients.

## Conclusions

Our study found a high susceptibility of HD patients both in the first and the second wave of COVID-19 compared to the general population. Patients in the incubation period may be the primary source of COVID-19 spread within the HD center. Disease symptoms, radiological findings, and laboratory tests were similar in both waves and in general. Patients with age > 60 years had more severe disease and a high mortality rate in both waves. Mortality in HD patients was higher in both waves than in the general population. The number of patients who developed critical disease and required hospitalization and mechanical ventilation was higher in the second wave. Death associated with COVID-19 in HD patients was 1.46 and 1.54 times higher than in the general population in the first and second waves. A high susceptibility and poor outcome in both waves of COVID-19 suggest that we are still not fully prepared and need more experience and better management policies for HD patients.
